# Enormous Hydrocele

**Published:** 1913-04

**Authors:** 


Enormous Hydrocele. H. G. Henrikson of New Market, Minn., *Am. Jour. of Clin. Med.,* Dec., 1912, reports a case in a man of 75, so large that when the tumor was first exposed he thought it was the abdomen, the penis lying in a dimple that resembled the umbilicus. About a gallon of liquid was removed, without injection of carbolic acid or other substance to prevent reaccumulation, but there has been no recurrence. Van Buren & Keyes record a hydrocele containing two quarts, and Dujat of Calcutta eighteen cases varying from 50 to 120 ounces.

